# Adjuvant Chemoradiotherapy for Gastric Cancer: Efficacy and Cost-Effectiveness Analysis

**DOI:** 10.3389/fonc.2019.01357

**Published:** 2019-12-03

**Authors:** Mengxi Zhang, Feng Wen, Xiaofeng He, Weihan Zhang, Jiankun Hu, Qiu Li

**Affiliations:** ^1^Department of Medical Oncology, Cancer Center, West China Hospital, Sichuan University, Chengdu, China; ^2^West China Biomedical Big Data Center, Sichuan University, Chengdu, China; ^3^Department of Medical Oncology, The First People's Hospital of Longquanyi District, Chengdu, China; ^4^Department of Gastrointestinal Surgery, West China Hospital, Sichuan University, Chengdu, China

**Keywords:** cost-effectiveness, gastric cancer, D2-gastrectomy, adjuvant chemotherapy, adjuvant chemoradiotherapy

## Abstract

**Purpose:** The benefit of adjuvant chemotherapy (CT) for localized gastric cancer (GC) after D2-gastrectomy has been clearly demonstrated. However, adjuvant chemoradiotherapy (CRT) remains controversial. This study aimed to assess the efficacy and cost-effectiveness of treatment for GC after D2-gastrectomy.

**Materials and methods:** Stage IB–IIICGC patients who had received adjuvant CRT or CT, or who had just been observed after D2-gastrectomy were retrospectively selected. Therapeutic strategy after surgery, disease-free survival (DFS), overall survival (OS), adverse events and costs were recorded retrospectively. A Markov model was developed to simulate the process of GC after D2-gastrectomy. Health outcomes were measured using quality-adjusted life-years (QALYs). Incremental cost-effectiveness ratio (ICER) was regarded as the primary outcome.

**Results:** A total of 254 patients were selected. Three year OS and DFS were 83.02 and 64.15% in the adjuvant CRT group, 74.19 and 63.54% in the adjuvant CT group, and 45.45 and 43.35% in the observation group. Total grade 3 or 4 toxicity was higher in the CRT group than in the CT group (54.72% vs. 37.10%, *p* < 0.05). The ICER of the CT and CRT groups vs. the observation group were $10,571.55 and $11,467.41/QALY, respectively. The probability of CT, CRT and observation being cost-effective were 28.9, 37.9, and 33.2%, respectively, when a willingness-to-pay threshold (WTP) of $25,648.45/QALY was used.

**Conclusions:** Adjuvant CRT was associated with improved OS and DFS compared with adjuvant CT and postoperative observation. Both adjuvant CRT and CT are likely to be cost effective compared with postoperative observation. However, adjuvant CRT was the optimal choice for a WTP threshold of $25,648.45/QALY.

## Introduction

Gastric cancer (GC) is the fifth most common malignancy and the third leading cause of cancer death worldwide. GC remains among the leading causes of global cancer burden (1). Eastern Asia, Eastern Europe and South America are major endemic regions with a high incidence of gastric cancer. There were 679,100 new cases and 498,000 deaths in China, accounting for more than 60% of the total morbidity and mortality worldwide (2). Currently, D2-gastrectomy has been widely considered as the best potentially curative treatment for patients with localized GC and is the standard procedure in Asia. However, even when curative resection (R0) is possible, recurrence still ranges from 32 to 41.7% (3–5), indicating that the effectiveness of surgery alone remains poor and unsatisfactory.

As an important component of resectable GC therapy, adjuvant chemotherapy (CT) results in increased median survival. Two pivotal Phase III trials, adjuvant CT combined with the drug S-1 in the ACTS-GC trial and capecitabine and oxaliplatin (XELOX) in the CLASSIC trial, have demonstrated that adjuvant CT reduces the risk of relapse and death in patients with GC after D2 lymphadenectomy (3, 4, 6). Both S-1 and XELOX treatments were recommended as adjuvant therapy regimens in the 2012 National Comprehensive Cancer Network clinical practice guidelines. In spite of this, adjuvant chemotherapy after D2-gastrectomy has not resulted in favorable prognosis, with recurrence observed in 23 to 30.6% of patients (3, 4, 6). The frequency of such reoccurrence suggests that radiotherapy is an attractive option for adjuvant therapy.

Through the use of adjuvant 5-fluorouracil/folinic acid (5-FU/FA) with 45 Gy locoregional irradiation, the pivotal intergroup study 0116 (INT-0116) demonstrated significant improvements in overall survival (OS) and disease-free survival (DFS) in the first randomized setting (7), but was criticized for 90% of patients receiving D0 or D1 lymphadenectomy. The ARTIST trial was conducted to further explore the efficacy of adjuvant chemoradiotherapy (CRT) following D2-gastrectomy. The result of this trial indicated that for patients with GC who underwent D2 lymph node dissection, CRT or CT did not provide increased DFS or OS but found a significant increase in 3 year DFS in patients with lymph node-positive disease after subgroup analysis (8).

Despite wide acceptance of adjuvant therapy, there has been no comparison of therapeutic effect between adjuvant CT and CRT, particularly in countries in which D2 resection is a routine surgical procedure (9). In addition, increasingly effective regimens are invariably associated with higher costs and toxicity profiles which must be weighed against their potential long-term benefits so that an informed choice of which therapy should be used to target the disease. Therefore, it is meaningful to explore whether radiotherapy combined with adjuvant CT will further improve survival in addition to cost-effectiveness. This study retrospectively analyzed not only the efficacy but also the cost-effectiveness of adjuvant CT and adjuvant RCT in GC patients from a Chinese societal perspective.

## Materials and Methods

### Patients and Treatment Regimens

Patients were retrospectively identified from the medical records of West China Hospital, Sichuan University, People's Republic of China, from November 2010 to October 2016. The patient inclusion criteria were as follows: (1) Histologically proven gastric or gastroesophageal adenocarcinoma. (2) Received D2 R0 gastrectomy. (3) Stage IB (with high-risk features: poorly differentiated, lymphovascular invasion, neural invasion or <50 years of age) to IIIC according to the American Joint Committee on Cancer (AJCC) TNM 7th Edition Staging. (4) Age ≥ 18 years. Patients who had received neoadjuvant therapy, were suffering with a second malignancy or had incomplete medical records were excluded. Therapeutic strategy following surgery, DFS, OS, treatment adherence, adverse events (AEs) and costs were recorded. OS was defined as the time from surgery to death, or the end of follow-up and DFS was defined as the time from surgery to disease recurrence, as confirmed by using imaging. Treatment adherence was defined as the percentage of patients who completed planned therapy.

The assessed treatments were grounded on the following schedules for administration of CRT or CT strategies to patients with DFS after D2-gastrectomy. In the CT group, patients received 1 year of S-1, or half year of capecitabine, or four to six cycles of the XELOX regimen (capecitabine plus oxaliplatin), or four to six cycles of the SOX regimen (S-1 plus oxaliplatin), or 8 to 12 cycles of FOLFOX (fluorouracil, leucovorin, plus oxaliplatin), or four to six cycles paclitaxel-based agents, according to clinical guidelines.

In the CRT group, a contrast-enhanced computed tomography scan with 3 mm-thick slices was conducted from the top of the diaphragm to the bottom of L4. Radiation fields were targeted to the anastomosis site, duodenal stump, tumor bed, and regional nodal coverage depending on the location of the primary disease, and 2 cm beyond the proximal and distal margins of the resection. The tumor bed was not irradiated except for T4 lesions. Organs at risk (small bowel, spinal cord, kidneys and liver) were delineated. All cases received intensity-modulated radiation therapy (IMRT) after the first or second cycle of the chemotherapy. The prescription dose was 45–50.4 Gy, with 1.8 Gy daily fractions administered over 5–5.6 weeks. Then the patients separately followed by the rest cycle of chemotherapy, according to clinical guidelines.

### Evaluation and Follow-Up

Sites of relapse were coded as loco-regional if a tumor was detected at the site of anastomosis, remnant stomach, or tumor bed, or regional lymph nodes within the radiation fields, whereas other recurrences outside the scope of radiation fields, liver metastasis, peritoneal seeding, or metastasis of other extra-abdominal sites were classified as “distant recurrence.” Regular follow-up theoretically appointments for all groups of patients entailed a physical examination, liver function test, complete blood count, chest radiography and abdominal computed tomography. In addition, gastroscopy was conducted annually or when clinically indicated. For the first 2 years, follow-up visits were scheduled at 3 month intervals, then every 6 months interval for the following 3 years and at 1 year intervals for the remaining life of the patient.

Adverse events were graded according to the National Cancer Institute's Common Terminology Criteria for Adverse Events (version 4.0).

### Model Structure

Decision analysis with Markov modeling was conducted using RStudio software (version 1.2.1335; 250 Northern Ave, Boston, MA 02210) and TreeAge Pro 2011 (TreeAge, Williamstown, Massachusetts, USA), to evaluate lifetime direct medical costs and health benefits associated with the three treatments of localized gastric cancer after D2-gastrectomy, based on the medical records of West China Hospital, including 3 mutually exclusive states: DFS, disease recurrence and death. The cohort proceeded from the end of D2-gastrectomy to death. All patients initially entered the model in the DFS state and either remained in the original state or moved to one of the other states at the end of each model cycle, based on the probability of transition between the two states. Weibull survival functions were then independently fitted to the reconstructed KM probabilities for both PFS and OS for the three treatments, with transition parameters and proportions based on clinical records, to the greatest possible extent (Table 1) (10). The probabilities of transition between health states were estimated based on an equation used previously: exp (–λ ^*^ (t + 1)^γ^ + λ ^*^ (t)^γ^), derived from the equations below: *P* (t→ t + 1) = [S(t) – S(t + 1)]/S(t) (11). Based on the transition probabilities estimated from DFS and OS, patients could switch to a different state at the end of each cycle in the Markov model. The model cycle length was 1 month, and the time horizon chosen for this analysis was a lifetime. Tracked costs, QALYs, ICER, comparison of adjuvant CT or adjuvant CRT vs. observation Model robustness were also explored using sensitivity analyses (12).

**Table 1:** Input parameters for the model.

**Parameters**	**Values**
**Weibull survival model of DFS**	
Adjuvant chemoradiotherapy	λ = 0.00159; γ = 1.71288
Adjuvant chemotherapy	λ = 0.00093; γ = 1.80379
Postoperative observation	λ = 0.00122; γ = 2.11482
**Weibull survival model of OS**	
Adjuvant chemoradiotherapy	λ = 0.00011; γ = 2.34675
Adjuvant chemotherapy	λ = 0.00052; γ = 1.83045
Postoperative observation	λ = 0.00051; γ = 2.11482

*λ: scale; γ: shape*.

This study was approved by the Research Ethics Committee of West China Hospital, Sichuan University.

### Cost and Resource Data

Direct costs included cost of drugs, radiation therapy, venous access management, nursing care, tests, hospitalization, and those for outpatient services and treatments for grade 3–4 AEs. Additionally, indirect costs of absenteeism from work were included. All costs were calculated from the Chinese societal perspective. The prices of chemotherapeutic agents were Chinese national drug prices. Unit costs of laboratory and radiological tests were retrieved from hospital accounting records. Detailed data on grade 3–4 AEs were derived from records of patients with localized gastric cancer after D2-gastrectomy at West China Hospital. Indirect costs were calculated as the number of days due to sickness multiplied by the minimum wage. Other costs, such as supportive care cost and expenses associated with travel to seek treatment, were not collected. All costs were converted to US dollars with an exchange rate of $1 = 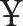
6.328 (21 March 2018).

### Effectiveness Data and Utility

Health outcomes were converted into quality-adjusted life year (QALY), and cost-effectiveness evaluated as an incremental cost-effectiveness ratio (ICER), which were calculated using the formula: (cost of strategy A—cost of strategy B)/(effectiveness of strategy A—effectiveness of strategy B). The willingness-to-pay (WTP) threshold in the analysis was set at $25,648.45/QALY (3 × per capita GDP of China, 2018) according to the World Health Organization guidelines for cost-effectiveness analysis (13–15). Costs and benefits in this study were discounted 3% annually. Health outcomes were denoted in gain in QALYs, and the utility scores of Markov states were obtained from previously published studies. Utility values were preference weights that can be used to quantify the quality of life (QOL) in each state. The mean utility values for remission after surgery and for metastasis were calculated to be 0.88 and 0.42, respectively (16). QALYs for individuals were estimated based on utility values.

### Sensitivity Analysis

The impact of essential variables on the results of the analysis was explored by one-way sensitivity analysis expressed as a tornado diagram. To investigate the uncertainty parameters, probabilistic sensitivity analysis was conducted based on a Monte Carlo simulation of 1,000 items. Additionally, a cost-effectiveness acceptability curve (CEAC) was performed.

## Results

### Patient Baseline Characteristics

A total of 254 patients were retrospectively identified from the records of West China Hospital, of which 169 were males. The median age was 59 years (range 22–84). Eastern Cooperative Oncology Group performance status for all patients received adjuvant treatment were 0 or 1. Baseline demographic and disease characteristics of the three strategies are presented in Table 2. No significant differences were observed in sex, patient status, pathological tumor stage or type of lymph node dissection and gastrectomy. Nevertheless, statistically older patients were enrolled in the observation group than the adjuvant treatment groups. Among all cases, 53 (21%) underwent adjuvant CRT and 124 (49%) underwent adjuvant chemotherapy. In the adjuvant CT group, 32 (25.81%) received a single-agent regimen (S-1 or capecitabine) and 92 (74.19%) received a two-drug cytotoxic regimen (based on SOX, XELOX, FOLFOX, or paclitaxel). For the adjuvant CRT group, 7.55% of cases received a single-agent regimen and 92.45% received a two-drug cytotoxic regimen with radiotherapy starting in the second cycle. The average duration of RT was 37.26 days (CI 95%; 35–42), and approximately 83.01% (44 of 53) or higher of cases completed the planned radiotherapy dose. Overall, treatment was completed as planned by 57.26% (71 of 124) of patients in the CT group and 66.04% of patients (35 of 53) in the CRT group. The median treatment lasted for 18 weeks (range of 3–56 weeks) for the former and 12 weeks (range of 3–47 weeks) for the latter. Although the adherence rate of CRT group was higher than the CT group, there was no statistical difference between the two groups (Table 3). After disease recurrence or metastasis, approximately 56.45% of patients in the CT group received salvage treatment, whereas 76.19 and 45.28% of patients in the CRT group and observation group received salvage treatment, respectively.

**Table 2:** Baseline demographic and clinical characteristics.

	**Adjuvant chemotherapy**	**Adjuvant chemoradiotherapy**	**Observation**
No.	124	53	77
Age (median)	56	50	62
**Sex—no. (%)**			
Male	81 (65.32)	32 (60.38)	56 (72.73)
Female	43 (34.68)	21 (39.62)	21 (27.27)
**ECOG performance status—no. (%)**			
0	63 (50.81)	28 (52.83)	28 (36.36)
1	58 (46.77)	24 (45.28)	43 (55.84)
≥2	0 (0)	0 (0)	4 (5.20)
Missing data	3 (2.42)	1 (1.89)	2 (2.60)
**AJCC stage (TNM classification)—no. (%)**			
IB	10 (8.06)	2 (3.77)	4 (5.19)
IIA	26 (20.97)	6 (11.32)	8 (10.39)
IIB	20 (16.13)	7 (13.21)	18 (23.38)
IIIA	23 (18.55)	15 (28.30)	17 (22.08)
IIIB	39 (31.45)	20 (37.74)	25 (32.47)
IIIC	6 (4.84)	3 (5.66)	5 (6.49)
**Primary tumor classification stage—no. (%)**			
T1	6 (4.83)	4 (7.55)	0 (0)
T2	21 (16.94)	9 (16.98)	14 (18.18)
T3	86 (69.35)	36 (67.92)	55 (71.43)
T4	11 (8.88)	4 (7.55)	8 (10.39)
**Regional lymph nodes classification—no. (%)**			
N0	23 (18.55)	2 (3.77)	12 (15.59)
N1	32 (25.81)	6 (11.32)	14 (18.18)
N2	23 (18.55)	19 (35.85)	16 (20.78)
N3	46 (37.09)	26 (49.06)	35 (45.45)
**Lauren classification—no. (%)**			
Intestinal	41 (33.06)	8 (15.09)	26 (33.77)
Diffuse	64 (51.61)	40 (75.47)	37 (48.05)
Mixed	19 (15.33)	5 (9.44)	14 (18.18)
**Adjuvant chemotherapy—no. (%)**			
S-1	20 (16.13)	2 (3.77)	–
Capecitabine	12 (9.68)	2 (3.77)	–
XELOX	9 (7.26)	1 (1.89)	–
SOX	37 (29.84)	29 (54.71)	-
FOLFOX	36 (29.03)	17 (33.08)	-
Paclitaxel-based agents	10 (8.06)	1 (1.89)	-
**3-year disease-free survival rate (95% CI)**	66.06 (56.87–73.75)	66.04 (51.64–77.06)	44.71 (33.34–55.43)
Number of disease recurrence events**—**no. (%)	62 (50.00)	21 (39.62)	53 (68.83)
Number of patients received salvage treatments**—**no. (%)	35 (56.45)	16 (76.19)	24 (45.28)

Data are expressed as number (%) or rate (95% CI) as appropriate.

*XELOX, Capecitabine + Oxaliplatin; SOX, S-1 + Oxaliplatin; FOLFOX, Oxaliplatin + 5-Fu; Paclitaxel-based agents, Paclitaxel + Oxaliplatin, Paclitaxel + Cisplatin, Paclitaxel + 5-Fu; ECOG, Eastern Cooperative Oncology Group*.

**Table 3:** Treatment adherence, according to adjuvant strategy.

	**Adjuvant chemotherapy**	**Adjuvant chemoradiotherapy**	***P*-value**
*N*	124	53	
**Therapy duration**			
Median (IQR)—wk	18 (12–18)	12 (12–12)	
Simple range—wk	3–56	3–47	
Missing data—no. (%)	7 (5.6)	2 (3.7)	
**Completion of cycles—no. (%)**			
Received scheduled no. of cycles	71 (57.3)	35 (66.0)	0.275
Did not receive schedule no. of cycles	31 (25.0)	9 (17.0)	
Received more than scheduled no. of cycles	10 (8.0)	3 (5.7)	
Missing data	12 (9.7)	6 (11.3)	

*IQR, denotes interquartile range; wk, week*.

### Safety

Toxicity experienced during treatment is listed in Table 4. The incidence of AEs greater than grade 3 in the CRT group was significantly higher than in the CT group. Gastrointestinal and hematological toxicities predominated. The most frequent drug-related grade three or four AE was leukopenia (12.9%) and nausea (13.71%) for the CT group, and leukopenia (28.30%), nausea (13.2%) and thrombocytopenia (5.66%) for the CRT group. Few patients experienced grade 3 hand–foot syndrome or neurosensory toxicity. AEs that led to dose reduction or treatment delay occurred in 37.1% of patients (46 of 124) in the CT group and 54.7% (29 of 53) in the CRT group. After treatment modification (delay or dose reduction) in addition to symptomatic treatment, the majority of occurrences of grade three or four AEs recovered. No treatment-related deaths occurred.

**Table 4:** Adverse events used in the decision model, according to treatment therapy.

	**Adjuvant chemotherapy**	**Adjuvant chemoradiotherapy**
No.	124	53
**Adverse events more than grade 3—no. (%)**		
Neutropenia	16 (12.9)	15 (28.30)
Nausea/vomiting	17 (13.71)	7 (13.20)
Thrombocytopenia	2 (1.61)	3 (5.66)
Stomatitis	3 (2.41)	1 (1.89)
Decreased hemoglobin	3 (2.41)	1 (1.89)
Elevated ALT/AST level	2 (1.61)	1 (1.89)
Hand**—**foot syndrome	2 (1.61)	1 (1.89)
Neurosensory toxicity	1 (0.81)	0 (0)
Total adverse events more than grade 3**—**no. (%)	46 (37.10)	29 (54.72)

### Efficacy

**Figure 1:**
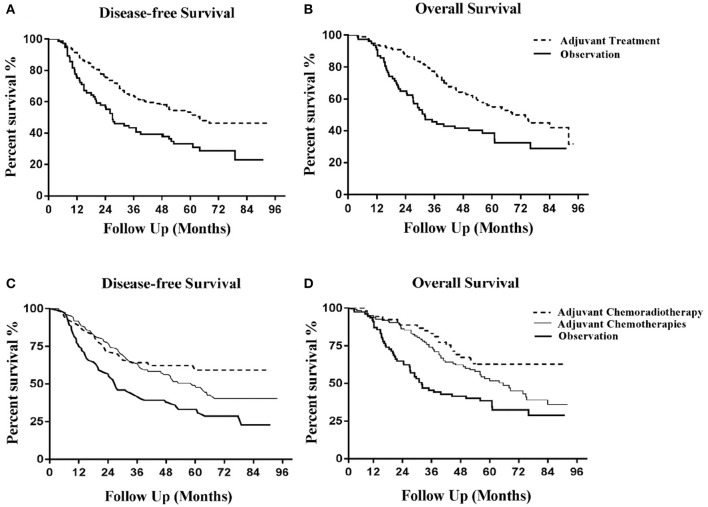
Kaplan-Meier curves of disease-free survival and overall survival. **(A)** Disease-free survival for adjuvant treatment and observation groups. **(B)** Overall survival for adjuvant treatment and observation groups. **(C)** Disease-free survival for adjuvant chemoradiotherapy, adjuvant chemotherapy and observation groups. **(D)** Overall survival for adjuvant chemoradiotherapy, adjuvant chemotherapy and observation groups.

After a median follow-up duration of 45.7 months (range 3.8–93.9), 136 disease recurrence events (62/124 in the adjuvant CT group, 21/53 in the adjuvant CRT group and 53/77 in the observation group) and 133 deaths (63/124 in the adjuvant CT group, 19/53 in the adjuvant CRT group and 51/77 in the observation group) were documented. The 3 year DFS and OS rates were higher in the adjuvant treatment group (adjuvant CT or adjuvant CRT group) than in the observation group. Kaplan-Meier curves for DFS demonstrated early separation between the two groups (Figure 1). After adjustment, the HR estimates for adjuvant-treated patients compared with patients in the observation group were 0.47 (95% CI: 0.32–0.70; *P* = 0.0002) for OS and 0.54 (95% CI: 0.37–0.79; *P* = 0.0002) for DFS. These values (Figures 1A,B) indicate a relative risk reduction in patients receiving adjuvant therapy of 53% for OS and 46% for DFS. However, model-derived survival curves for DFS did not differ significantly between the CRT and CT groups (HR: 0.73; 95% CI; 0.46–1.15; *P* = 0.20), in addition to OS (HR: 0.65; 95% CI: 0.41–1.03; *P* = 0.17) (Figures 1C,D). Median PFS and OS have not yet been reached for the adjuvant CRT group. For the CT, CRT and observation groups, the rates of 3 year OS were 74.19, 83.02, and 45.45%, respectively, and 63.54, 64.15, and 43.35% for DFS, respectively.

### Cost-Effectiveness Analysis

According to this study, treatment with CT provided an effectiveness gain of 5.05 QALYs at a mean cost of $25,878.04. The CRT group gained 6.86 QALYs per patient at a mean cost of $47,942.01 per patient over a lifetime (3% discounted rate for both effect and costs). Patients in the observation group had 3.59 QALYs at a mean cost of $10,443.57. Together, the ICER of the adjuvant CT group and adjuvant CRT group vs. the observation group was $10,571.55/QALY and $11,467.41/QALY, respectively (Table 5).

**Table 5:** Cost, utility and base case analysis of the decision model.

**Cost item ($) per patient per month**	**Mean base-case value**		
	**Adjuvant chemotherapy**	**Adjuvant chemoradiotherapy**	**Observation**
**Direct costs**			
Chemotherapy	166.87	0	0
Chemoradiotherapy	0	281.49	0
Hospital bed cost	0.64	0.52	0
Tests	93.87	90.75	92.14
Venous access	44.12	45.09	0
Nursing care	7.40	6.25	0
Outpatient	4.7	4.7	4.7
Total direct cost	317.6	428.8	88.93
Grade 3–4 AE-related cost	3.34	3.71	0
Cost of time loss	23.78	30.43	8.91
Cost of the disease-free state per patient per month	344.73	462.94	97.84
Cost after recurrence per month per patient per month	468.95	503.98	514.70
**Lifetime cost**			
Cost of DFS	22627.12	39610.62	4228.04
Cost of disease recurrence	3250.92	8331.39	6215.54
Total cost	25878.04	47942.01	10443.57
Incremental cost^*^	15434.47	37498.44	
**Effectiveness**			
Effectiveness for the DFS	4.81	6.28	3.17
Effectiveness for the disease recurrence	0.24	0.58	0.42
Total effectiveness (QALYs)	5.05	6.86	3.59
Incremental effectiveness^*^	1.46	3.27	-
Incremental cost per QALY (ICER)^*^	10571.55	11467.41	-
**Utility**			
DFS	0.88	0.88	0.88
disease recurrence	0.42	0.42	0.42
Death	0	0	0

* Compared with observation group.

*AE, adverse event; DFS, disease-free survival; DR, disease recurrence; ICER, incremental cost-effectiveness ratio; QALY, quality-adjusted life year*.

### Sensitivity Analysis

**Figure 2:**
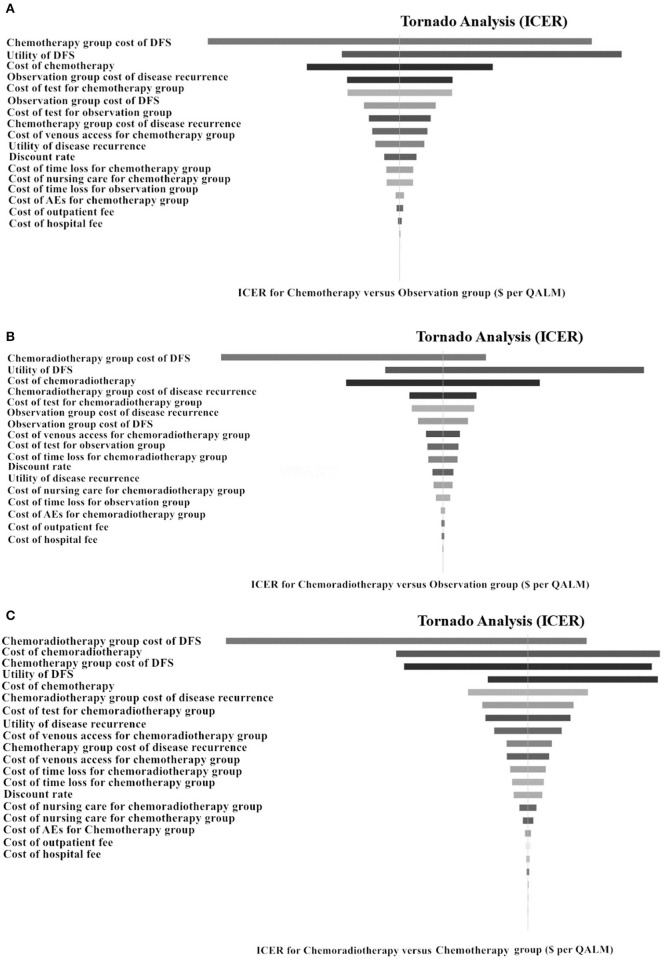
Tornado diagrams of 1-way sensitivity analyses for ICER. Tornado diagrams of univariate analyses for localized gastric cancer after D2-gastrectomy. **(A)** Adjuvant chemotherapy vs. observation. **(B)** Adjuvant chemoradiotherapy vs. observation. **(C)** Adjuvant chemotherapy vs. adjuvant chemoradiotherapy. These diagrams present the results of 1-way analyses of the parameters for 3 different strategies. The width of the bars represents the range of results of our analysis when the parameters are changed. p, transition probability; DFS, disease-free survival; QALM, quality-adjusted life month (quality-adjusted life year/12); ICER, incremental cost-effectiveness ratio.

To investigate the impact of the most influential variables on the results, a one-way sensitivity analysis was conducted by varying the model parameters over their range of values (±30%). The results of the analysis are displayed in Figure 2 as tornado diagrams. Model parameters with a substantial impact on the results of cost-effectiveness analysis are presented in order. In the first analysis, the cost-effectiveness of the CT group vs. the observation group was sensitive to the monthly cost value of DFS in the CT group (Figure 2A). As the value ranged from our baseline estimate of $241.31 to $488.15, the ICER increased significantly from $5,827.24 to $15,084.12 per QALY gained. In the second analysis, the monthly cost value of DFS in the CRT group had the strongest impact on the results (Figure 2B). For values ranging from $265.5 to $500.5, the ICER changed from $6,490.56 to $12,549.12 per QALY gained. Additionally, the robustness of the ICER of CT vs. CRT was tested (Figure 2C). ICER was sensitive to the monthly cost of DFS in the CRT group. For values ranging from a baseline estimate of $241.31 to $488.15, the ICER changed from $3,112.44 to $6,082.96 per QALY gained. The costs of time lost, venous access, outpatient fee or inpatient fee had little impact on the robustness of the three cost-effectiveness analyses above.

**Figure 3:**
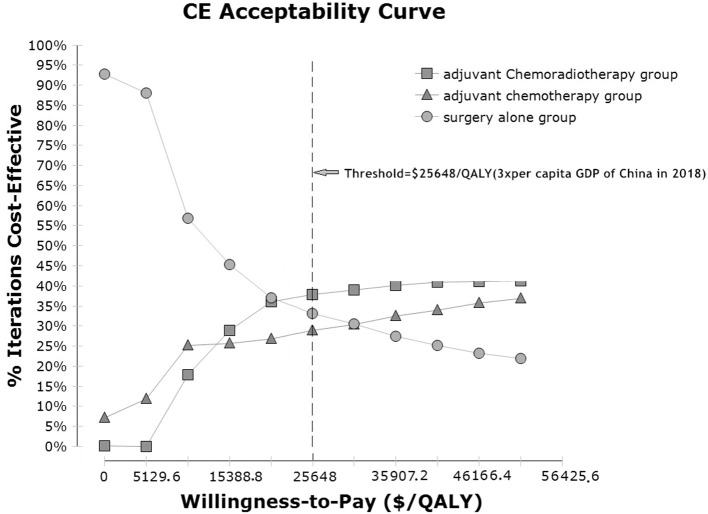
Cost-effectiveness acceptability curves for the three adjuvant strategies in localized gastric cancer after D2-gastrectomy. Cost-effectiveness probabilistic acceptability curves showing the probabilities of acceptability of each strategy for different WTP thresholds. The horizontal axes represent willingness-to-pay thresholds to gain 1 additional QALM. Three curves are presented for the adjuvant chemotherapy group, adjuvant chemoradiotherapy group, and observation group. CE, cost-effectiveness; QALM, Baseline demographic and clinical characteristics quality-adjusted life month; GDP: gross domestic product.

In a probabilistic sensitivity analysis based on a Monte Carlo simulation of 1,000 items, the CEAC revealed the preferred strategies when accounting for a range of cost-per-QALY thresholds. The results of the analysis are presented in cost-effectiveness acceptability curves (Figure 3). The probabilities of the CT, CRT and observation groups being cost-effective were 28.9, 37.9, and 33.2%, respectively, using a WTP threshold of $25,648.45/QALY.

## Discussion

In this retrospective analysis, we demonstrated increased survival and well-tolerated toxicity for adjuvant CT with or without concurrent radiotherapy in patients with stage IB to IIIC CG after D2-gastrectomy compared with postoperative observation. In addition, this study demonstrated marginal benefits in terms of OS or DFS in patients treated with adjuvant CRT relative to those treated by adjuvant CT, although this was not statistically significant. Rates of compliance in this study were similar to the rate of 64% observed in the experimental group of the INT-0116 trial, with treatment completed as planned in 57.26% (71 of 124) of patients in the CT group and 66.04% of patients (35 of 53) in the CRT group.

In comparison to the literature, the survival benefit at 3 years in the present study (OS 74%, DFS 66%) for the CT group was lower than that observed in the CLASSIC study (OS 85%, DFS 84%) and ACTS-GC trial (OS 80.1%, DFS 72.4%) (3, 4). The principal reason may be that the proportion of early stage patients (45% in stage IB–II) in this study was lower than the CLASSIC and ACTS-GC trials, with 49.7 and 64%, respectively. For the CRT group, survival rates (OS 83.02%, DFS 64.15%) were more similar to those observed in the ARTIST trial (OS 78.2%, DFS 74.2%) but better than the results of Intergroup 0116 (OS 50%, DFS 41%) (7). This difference may be explained as a result of the lower proportion of patients who underwent D2-gastrectomy in the Intergroup 0116 since all cases in the present study received D2-gastrectomy. In addition, for some patients (9/124) the total radiation dose in this study (50.4 Gy/28f) was also higher than that in ARTIST (45 Gy/25f). Nevertheless, when comparisons were performed within treatment groups (adjuvant CT or CRT), the model-derived survival curves did not differ significantly between adjuvant CT and CRT, consistent with the randomized controlled ARTIST trial, although subgroup analysis suggested that radiotherapy might be beneficial for lymph-node-positive patients. This is understandable in view of a recently-published report of interim results in the ARTIST trial demonstrating that no difference in DFS between CT and CRT was observed (HR 0.910, *P* = 0.667) in the whole population, while subgroup analysis showed a benefit in PFS of combination radiotherapy with SOX in pN3 (73% 3 year DFS).

From safety analysis, the majority of adverse effects were grade 1 or 2. The total number of grade 3/4 toxic events was higher in the CRT group compared with the CT group (54.72% vs. 37.10%, *p* < 0.05). The most common non-hematologic grade 3 to 4 adverse events were vomiting, stomatitis and HFS, each of which occurred in 1.61–13.71% of patients in both groups. Grade 3 to 4 neutropenia occurred in 28.3% of patients in the CRT group and 12.9% in the CT group (Table 4), values consistent with the literature (7, 8). We anticipate with interest the ongoing prospective phase III trial (ChiCTR-TRC-12002919) in this hospital which is testing adjuvant CT vs. CRT in patients with localized GC after D2-gastrectomy (17).

The widespread use of adjuvant treatments has caused substantial economic burden. A number of studies have previously evaluated the economic implications of adjuvant strategies in the treatment of stage II-IIIB GC patients undergoing D2-gastrectomy, which have suggested that various adjuvant CT or CRT regimes were favorable in terms of long-term cost-effectiveness in contrast to D2-gastrectomy alone (16, 18–23). A report by Hisashige et al. (19) suggested that S−1 as an adjuvant strategy is cost-effective over a lifetime for curatively resected GC compared with surgery alone, with an ICER estimated to be $3,016 per QALY, using the results of the ACTS-GC trial. Similarly, Wang et al. (20) utilized the effectiveness of the Intergroup 0116 trial, and noted that at an ICER of $38,400/QALY when adjuvant chemoradiotherapy was used appears well below the WTP for malignancy and therefore in accordance with Western studies. Wu et al. (16) stated that adjuvant therapy with capecitabine plus oxaliplatin is a more cost-effective strategy than the S-1 strategy in stage II or III GC patients who have undergone D2 gastrectomy. However, these evaluations were conducted only in clinical trials or specific pair-wise comparisons within adjuvant CT, and not using patient-level data in real-world clinical practice. No economic assessment has compared all potential adjuvant treatment strategies after D2-gastrectomy from a holistic perspective.

According to this study, both the CT and adjuvant CRT groups produced greater QALY gains compared with the observation group but also at a greater cost. Our results revealed that adjuvant CT improved the effectiveness of localized gastric cancer after D2-gastrectomy by 1.46 QALYs compared with the observation group (5.05 QALYs vs. 3.59 QALYs) with an incremental cost of $15,434.47 ($25,878.04 vs. $10,443.57). Adjuvant CRT improved the effectiveness by 3.27 QALYs compared with observation (6.86 QALYs vs. 3.59 QALYs) with an incremental cost of $37,498.44 ($47,942.01 vs. $10,443.57). Both adjuvant CRT and CT are likely to be cost effective compared with postoperative observation. For WTP thresholds selected to be three times GDP of China, that is $25,648.45/QALY, adjuvant CRT was the optimal cost-effective choice.

Several limitations of the current analysis should be addressed. Firstly, this study was single-center and may not be representative of other regions of China. In addition, different strategies might be optimal for decision-makers in other countries with different WTP thresholds. However, in this study there was consistent surgical technique and one-way sensitivity analyses were conducted by varying the model parameters over their range of values (±30%) to represent conditions in different nations. Secondly, as the data in our study were collected retrospectively from medical records, some data (e.g., AEs, costs of treatment and DFS) might not be strictly accurate. In addition, this study included a broad variety of chemotherapy regimens which might have influenced the robustness of the results to a certain extent.

To the best of our knowledge, this study is the first real-world analysis to evaluate the efficacy and cost-effectiveness of CRT in patients with localized gastric cancer after D2-gastrectomy. We demonstrated that both adjuvant CRT and CT are likely to be cost effective compared with postoperative observation. Adjuvant CRT was the optimal choice for a WTP threshold of $25,648.45/QALY. We anticipate prospective studies to further confirm this conclusion.

## Data Availability Statement

All datasets generated for this study are included in the article/supplementary material.

## Ethics Statement

The studies involving human participants were reviewed and approved by the Research Ethics Committee of West China Hospital, Sichuan University. The patients/participants provided their written informed consent to participate in this study.

## Author Contributions

MZ and QL designed the study. MZ, XH, WZ, and JH collected the data. MZ, XH, and FW analyzed and interpreted the data. MZ, XH, and FW prepared the manuscript. All the authors read and approved the final manuscript.

### Conflict of Interest

The authors declare that the research was conducted in the absence of any commercial or financial relationships that could be construed as a potential conflict of interest.
